# Is It Possible to Combine Non-Invasive Brain Stimulation and Evidence-Based Psychosocial Interventions in Schizophrenia? A Critical Review

**DOI:** 10.3390/brainsci14111067

**Published:** 2024-10-26

**Authors:** Jacopo Lisoni, Gabriele Nibbio, Antonio Baglioni, Simona Dini, Bianca Manera, Alessandra Maccari, Luca Altieri, Irene Calzavara-Pinton, Andrea Zucchetti, Giacomo Deste, Stefano Barlati, Antonio Vita

**Affiliations:** 1 Department of Mental Health and Addiction Services, ASST Spedali Civili of Brescia, 25123 Brescia, Italy; irenecalzavarapinton@gmail.com (I.C.-P.); andreazucchetti@hotmail.it (A.Z.); stefano.barlati@unibs.it (S.B.); antonio.vita@unibs.it (A.V.); 2 Department of Clinical and Experimental Sciences, University of Brescia, 25123 Brescia, Italy; gabriele.nibbio@gmail.com (G.N.); a.baglioni@unibs.it (A.B.); s.dini@unibs.it (S.D.); b.manera@unibs.it (B.M.); a.maccari@unibs.it (A.M.); l.altieri@unibs.it (L.A.); giacomodeste@mac.com (G.D.); 3 Department of Mental Health and Addiction Services, ASST Vallecamonica, 25040 Brescia, Italy

**Keywords:** NIBS, psychosocial interventions, integrated treatment, negative symptoms, cognitive impairment, tDCS, rTMS, iTBS, cognitive remediation

## Abstract

In schizophrenia, it was suggested that an integrated and multimodal approach, combining pharmacological and non-pharmacological interventions, could improve functional outcomes and clinical features in patients living with schizophrenia (PLWS). Among these alternatives, evidence-based psychosocial interventions (EBPIs) and Non-Invasive Brain Stimulation (NIBS) represent feasible treatment options targeting the clinical features that are unmet needs of PLWS (especially negative and cognitive symptoms). As no clear evidence is available on the combination of these non-pharmacological approaches, this review aimed to collect the available literature on the combination of EBPIs and NIBS in the treatment of PLWS. We demonstrated that the field of combining EBPIs and NIBS in schizophrenia is in its infancy, as only 11 studies were reviewed. In fact, only a few trials, with divergent results, combined these non-pharmacological modalities; while emerging evidence is available on the combination of cognitive remediation and rTMS/iTBS, inconclusive results were obtained. Conversely, albeit preliminary, more solid findings are available on the combination of HF-rTMS and family intervention. Moreover, despite the fact that cognitive activation could not be considered an EBPI, promising results are available in combination with tDCS to improve the working memory domain. To overcome these limitations, we considered several methodological issues to promote research in this field.

## 1. Introduction

### 1.1. Current Treatment Options for Schizophrenia

Schizophrenia represents a leading cause of disability worldwide as this mental disorder is characterized by a multi-episodic course and chronic progression in up to 60% of cases [1]. Moreover, schizophrenia is responsible for massive societal and economic costs with great impacts on patients, relatives, and the organization of mental health services [2,3,4,5]. Indeed, patients living with schizophrenia (PLWS) present impaired real-world functional outcomes [6,7], reduced quality of life [8], high levels of internalized stigma [9], and low levels of life engagement [10].

Despite antipsychotics being effective at reducing acute psychotic and disorganized symptoms [11], the treatment of this mental condition is not yet completely resolved. Indeed, while current findings showed that the response to antipsychotics is commonly incomplete so that between 20% and 30% of PLWS are resistant to treatment [12], it was also clear that most patients, even those with a good response to medications, continue to experience disabling residual symptoms, and impaired social and occupational functioning [1,2]. Furthermore, the effectiveness of antipsychotics on the clinical dimensions mostly associated with detrimental functional outcomes [13], such as negative and cognitive symptoms, is not complete [14,15,16,17,18]. Additionally, current evidence also highlighted that antipsychotics only modestly improve the psychosocial functioning and the quality of life of PLWS [18,19,20,21]. Another aspect related to antipsychotics is that these drugs, especially the second-generation antipsychotic (SGA), are responsible for metabolic adverse effects, including dyslipidemia, obesity, and type 2 diabetes, which in turn have a negative impact on cognitive performance and well-being [22,23]. Thus, as the relevance of the adverse effects phenomenon has severe implications on medication adherence [24], it was suggested that the choice of antipsychotics must be personalized and guided according to their tolerability and safety profile, particularly considering the anticholinergic, histaminergic, and metabolic burdens [11,25].

Thus, although pharmacotherapy remains the mainstay to achieve and maintain symptomatic remission, the treatment of schizophrenia is not limited to symptom control; it should be guided by the need to achieve recovery [26,27]. Recovery is a multidimensional concept that changed the paradigm in the treatment of schizophrenia, moving from an approach based mostly on symptom control to a model based on two main aspects: remission (a reduction or even absence of symptoms so that they do not interfere significantly with behaviors) and social functioning (defined as the capacity to function in the community socially and vocationally) [28]. In this context, the treatment of schizophrenia requires an integrated and multifaceted approach involving the combination of pharmacological and non-pharmacological interventions to effectively improve functional outcomes [26,27]. This is especially true for what concerns the treatment of two core domains of schizophrenia: on one hand, negative symptoms as this dimension represents a clinical unmet need in the care of PLWS [29]; on the other hand, neurocognitive and social cognitive domains, as these represent the clinical features that negatively impact functional outcomes the most, including impaired functional capacity and community functioning [13,30], social and interpersonal skills [31,32], and quality of life [32].

Currently, the recent European Psychiatric Association (EPA) guidelines on the treatment of negative and cognitive symptoms in PLWS strongly recommended to provide this integrated approach offering evidence on the effectiveness of some non-pharmacological interventions, namely evidence-based psychosocial interventions (EBPIs) and Non-Invasive Brain Stimulation (NIBS) interventions [14,15]. However, little is known about the possibility of combining these two approaches.

### 1.2. Evidence-Based Psychosocial Interventions (EBPIs)

As recovery is a journey aimed at achieving a meaningful life, improved quality of life, better physical health, social integration, and self-agency [26] promoting recovery, it is recommended to offer EBPIs [26,27,33,34,35]. Several EBPIs are available for PLWS, including Cognitive Remediation (CR), Meta-Cognitive Training for psychosis (MCT), Social Skill Training (SST), Psychoeducation (PE), Family interventions (FIs), Cognitive-Behavioral Therapies for psychosis (CBTp), Supported employment (SE), Assertive Community Treatment (ACT), and aerobic exercise (AE).

Through group/individual computerized/non-computerized sessions, CR is a behavioral training-based intervention targeting neurocognition and social cognition, producing durable improvements in psychosocial functioning [27,36]. Indeed, according to the EPA guidelines on the treatment of cognitive symptoms in schizophrenia, CR represents the EBPI with the highest degree of recommendation [14]. Enhancing metacognitive functions, MCT combines elements of psychoeducation, cognitive bias modification, and strategy teaching to target positive symptoms (specifically, delusions) and other persistent symptoms, providing significant long-term improvements in functional outcomes [27,37]. SST is aimed at targeting interpersonal and social skills to improve functional outcomes, including social performance and social interactions, and it also provides significant results on negative and general psychopathology symptoms [27,38]. PE incorporates several interventions focused on the education, on different themes, of an individual living with a psychiatric disorder to improve treatment and rehabilitation outcomes, allowing a behavioral change in the person [27]. For PLWS, PE was found to promote relapse prevention, treatment adherence, and psychosocial functioning [39]. By reducing the levels of family burdens, stress, and expressed emotions within families and enhancing the capacity of relatives to solve problems, FI usually includes psychoeducation interventions, producing significant effects on risks of relapse and of new hospitalizations, as well as improving compliance with medications [27,40]. CBTp is a structured adapted psychotherapy for PLWS, focused on the connections between thoughts, behaviors, and emotions in which the “here and now” focus allows the patient to develop skills to identify and address unhelpful thinking patterns and behaviors [27,39]. Several pieces of evidence highlighted CBTp’s effectiveness at improving clinical outcomes for PLWS, especially the severity of positive symptoms but also negative symptoms and functioning levels [39,41,42]. Moreover, CBTp showed positive effects on preventing the transition to full psychosis in patients at high risk [43]. ACT represents an intensive and person-centered mental health model with a multidisciplinary team approach aimed at improving clinical and functional outcomes, including helping with medications, housing, finances, and everyday problems in living [27,44]. Finally, physical exercise (particularly AE) could be considered for all intents and purposes as an EBPI for PLWS as it is able to improve not only physical fitness [45] but also psychopathological outcomes [46], cognitive performance [46,47], and functional outcomes [47].

While specific interventions are associated with the improvements of peculiar clinical features (i.e., cognitive domain enhancement following CR [48], negative symptoms reduction following SST [38], reduced stigma perception following psychoeducation [49], CBTp for positive symptoms [50]), it was also demonstrated that the integration between these EBPIs could further produce better symptomatologic and functional outcomes [39,42,51,52,53,54]. In fact, the EPA guidelines strongly recommended providing this integrated approach, offering evidence of the effectiveness of integrating these EBPIs. For example, for the treatment of negative symptoms, it was suggested that in addition to the preferable use of SGA, CR interventions, SST, and AE could be effective treatment options [14]. On the other hand, for the treatment of cognitive impairment, it was suggested to prefer SGA compared to first-generation antipsychotics for their favorable cognitive profile, whereas CR and AE were recommended as effective psychosocial interventions given their good-to-moderate level of scientific evidence [15].

### 1.3. Non-Invasive Brain Stimulation (NIBS)

For the first time ever, the EPA guidance on the treatment of cognitive impairment in PLWS observed that other non-pharmacological somatic interventions, namely NIBS, could be considered feasible add-on therapies [15]. In contrast, the EPA guidelines on the treatment of negative symptoms reported that, although NIBS was considered a promising intervention, the available evidence was not sufficient to provide general and definitive recommendations [14]. Nonetheless, these findings suggest how NIBS gained great scientific interest also in the case of schizophrenia. Indeed, despite its low incidence, this mental disorder represents the second most studied mental illness after major depressive disorder, as seen by the frequency of clinical studies using neuromodulation techniques conducted from the beginning of the new millennium up to now [55].

NIBSs are principally transcranial Direct Current Stimulation (tDCS) and repetitive Transcranial Magnetic Stimulation (rTMS). tDCS modulates cortical excitability, inducing polarity-dependent shifts of the neuronal membrane potential by delivering weak and constant currents (1–2 mA) through anodal and cathodal electrodes. Usually, anodal stimulation increases cortical excitability, whereas cathodal stimulation decreases it; however, the net effects depend on the impact on the global network balance [56,57,58]. Moreover, while tDCS’s effects also depend on several stimulation parameters, outlasting neuroplastic effects are explained by the calcium-dependent modulation of synaptic plasticity in N-methyl-D-aspartate (NMDA) glutamatergic neurons and of GABAergic activity through mechanisms similar to the long-term potentiation (LTP) and long term-depression (LTD) phenomena [57,58]. On the other hand, rTMS is based on the electromagnetic induction principle and consists of the delivery of localized electromagnetic pulses at certain frequencies to the cerebral cortex through a wire coil by inducing secondary electric current flows to induce action potentials in the neurons [59,60,61]. While the exact physiological bases of rTMS’s after-effects have not yet been clearly understood, it was suggested that the main mechanism underlying these after-effects resemble the LTP and LTD phenomena [61]. According to the frequency of delivered pulses, rTMS modalities consist of inhibitory (low-frequency rTMS, LF-rTMS; ≤1 Hz) or excitatory (high-frequency, HF-rTMS; >5 Hz) protocols, although other factors (including stimulation intensity, pulse number, and coil designs) are equally responsible for clinical after-effects [59,60]. When magnetic pulses are delivered in bursts of 3 trains at a frequency of 50 Hz with an inter-burst interval of 200 msec, the stimulation protocol is named Theta-Burst Stimulation (TBS). As TBS has several advantages over other conventional rTMSs due to its low intensity, short duration of application, and long-lasting effects, TBS has been used to mimic the brain’s natural theta rhythm occurring in the hippocampus to upregulate/downregulate the excitability of focal regions [61,62,63]. Two different patterns of TBS are commonly used: the intermittent TBS (iTBS) protocol consists of 2 s of TBS (30 pulses) repeated every 10 s for 190 s (600 pulses in total) to produce increased cortex excitability; the continuous TBS (cTBS) consists of either 300 pulses (20 s) or 600 pulses (40 s) delivered without any interruption leading to reduced cortical excitability [59,60,61,62,63].

When applied for the treatment of core symptoms of schizophrenia, NIBS targets two main brain areas. For the treatment of auditory verbal hallucinations (AVHs), since functional neuroimaging described hyperactivation of left-side auditory and linguistic areas of the brain (including the temporal-parietal junction and the left superior temporal cortex) as the neurobiological signatures of AVH [64,65], inhibitory NIBS (cathodal-tDCS or LF-rTMS) on these regions seem to improve AVH [66,67], especially when the tDCS was delivered as a twice-daily stimulation and with ≥10 stimulation sessions [67,68,69].

On the other hand, for the treatment of negative and cognitive symptoms, the main stimulated brain area with excitatory NIBS (anodal-tDCS or HF-rTMS/iTBS) [70,71] is the left Dorsolateral Prefrontal Cortex (DLPFC). Indeed, as frontal hypo-functioning [72,73], frontotemporal [73,74], and frontocortical-striatal networks impairments [73,75,76] represent the main neurobiological signatures involved in negative and cognitive symptoms pathophysiology; the left DLPFC is considered a central hub among these brain networks. Moreover, NIBSs are suggested to restore the aberrant connectivity and activity between these regions [77,78,79,80], also influencing GABAergic and glutamatergic transmissions [81,82,83]. Meta-analytic evidence found beneficial moderate improvements of negative symptoms with either DLPFC HF-rTMS/iTBS or anodal-DLPFC tDCS [70,71,84,85,86,87]. In detail, significant improvements were obtained when the stimulations consisted of ≥ 10 sessions [69,88], if tDCS was delivered twice daily [69,89], and if HF-rTMS was delivered at least at 10 Hz [90] for at least 3 weeks [71,90].

Conversely, NIBS’s effectiveness on cognitive symptoms of schizophrenia seems to be differential according to NIBS modalities. For tDCS, it is generally accepted that this intervention mainly improves the working memory [71,91,92,93,94] but also the attention [71,95] and social cognition domains [95]. However, these results are not definitive, as other findings showed no substantial cognitive improvement following tDCS [89,96,97,98]. Several reasons could explain these divergent results. While the variation of cognitive outcomes was usually assessed as a secondary outcome, a heterogenous evaluation of cognitive performance changes was usually performed in clinical studies [95]. Moreover, despite larger evidence suggesting stimulating the prefrontal cortex to improve cognitive functions, definitive recommendations on tDCS parameters are not currently available to ultimately enhance cognition in PLWS [95]. However, preliminary findings suggested that longer stimulation sessions could produce more favorable outcomes [97]. For rTMS/iTBS, the EPA guidance on the treatment of cognitive symptoms suggested that the available literature does not recommend rTMS/iTBS as an evidence-based treatment for cognitive impairment in PLWS according to the results of several meta-analyses [70,96,97,98,99,100]. Nevertheless, other meta-analysis reports demonstrated positive results mainly in the working memory domain [90,101,102] and, probably, in language abilities [90,101]. Moreover, while no definitive recommendation on the stimulation parameters to obtain a cognitive improvement following rTMS was achievable for PLWS, the main suggestions are to stimulate the left DLPFC [96,101] with a stimulation intensity of 20 Hz for a period longer than 1 month [96] and with total pulses <30.000 [101]. It is important to note that two meta-analyses, one on tDCS [94] and one on rTMS [101], found that more robust cognitive improvement in the working memory domain was achievable if clinical studies were designed with a congruent time of follow-up, leading to the conclusion that a time to reassess the variation of cognitive outcomes (e.g., a follow-up period) is necessary to consolidate the pro-cognitive effects of NIBS according to the biological mechanisms of action of these interventions (i.e., LTP phenomenon) [81,94,101].

Despite NIBS’s effectiveness at improving depressive symptoms in PLWS, despite positive results from RCTs using anodal prefrontal-tDCS [103,104], meta-analysis evidence provided divergent and inconclusive results [69,70].

Finally, considering whether NIBS could influence functional outcomes in PLWS, initial but inconclusive evidence is available. A suggestion to explain these negative results is that clinical studies usually did not evaluate NIBS’s effects on functional outcomes as primary aims. For example, while Dlabac-de Lange et al., 2015 [105] and de Jesus et al., 2011 [106] found no beneficial effect on the quality of life measures (QoL), respectively, with either 3 weeks of HF-rTMS (10 Hz) on bilateral DLPFC or 20 sessions of LF-rTMS (1 Hz) over the left temporal-parietal cortex, Kao et al., 2020 found significant improvements in the psychological domain of QoL following 10 twice-daily sessions of frontotemporal tDCS [107]. On the contrary, preliminary studies considered NIBS’s effects on functional capacity (FC) [104,108], the ability to perform tasks relevant to everyday life functioning in a structured and controlled environment guided by an examiner [109]. Assessed through the University of California, San Diego Performance-Based Skills Assessment (UPSA-B) [109], FC is an objective measurement of an individual’s ability to perform everyday living skills, social activities, and vocational abilities and represents a proxy of real-world functioning in PLWS [30,110,111,112]. Narita et al., 2017 [104] found that 10 sessions (twice-daily) of 2 mA prefrontal non-balanced tDCS (anode: left DLPFC; cathode: right orbitofrontal region) were effective at improving several FC measures at the UPSA-B with medium-to-large effect sizes at a 1-month follow-up. Subsequently, Yamada et al., 2023 confirmed that only 2 mA anodal tDCS on the left DLPFC, if compared to anodal stimulation of the left superior temporal sulcus, was associated with FC measure improvements. This confirmed that, on the one hand, electrode placement could influence NIBS’s efficacy at ameliorating functional outcomes in PLWS and, on the other, the relevance of the left DLPFC stimulation to improve such clinical features [108].

### 1.4. Aims

This critical review aimed to describe the available literature on the combination of EBPIs and NIBS in the treatment of PLWS. In doing so, we first offer an overview of these two approaches. Particularly, we informed of the greater relevance of the clinical effects of NIBS modalities in order to fill the lack of definitive guidelines on the treatment of schizophrenia through NIBS. By summarizing the available literature on the combination of EBPIs and NIBS in PLWS, we focused on well-known EBPI strategies, including cognitive remediation (CR) and family intervention (FI). Additionally, we offered an overview of the combination of NIBS with cognitive activation (CA) to improve cognitive impairments in PLWS. In the following pages, CA refers to the engagement in cognitive exercises repeatedly and with regular practice in order to foster the activation of specific brain loci and neural circuits. This approach is less focused on strategy coaching for cognitive exercises and more so on generalizing cognitive gains to real-world functioning that, on the other hand, represent core elements of CR. Indeed, in CR studies, the interventions go beyond simple cognitive activation and instead include an essential role of the active and trained therapist, who is tasked to help the participant in the development of novel cognitive strategies and facilitating the transfer of gains obtained in cognitive performance to everyday functioning. Finally, future directions on the feasibility and current limitations of combining NIBS and EBPIs are discussed.

## 2. Materials and Methods

### Search Strategy

The research topic was explored by searching relevant publications throughout several electronic databases (Scopus, PubMed, Web of Science, and Google Scholar) from database inception to 9 October 2024. The search strategy was based on the combination of several keywords: “schizophrenia” AND “brain stimulation” AND “psychosocial intervention” OR “cognitive remediation” OR “social skill training” OR “CBTp” OR “psychoeducation” OR “family intervention” OR “assertive community treatment” OR “meta-cognitive training for psychosis” OR “supported employment” OR “aerobic exercise” OR “cognitive activation” AND “tDCS” OR “rTMS” OR “TBS”. Reviews emerging from the search were also assessed to identify individual studies of interest. The available literature was analyzed according to the following inclusion criteria: papers published in peer-reviewed journals; available in English language only; no restrictions on study design were considered (RCTs, open-label, case reports); papers must have combined NIBS modalities (only tDCS, rTMS or TBS) with EBPI modalities (including cognitive remediation or social skill training or CBTp or psychoeducation or family intervention or assertive community treatment or meta-cognitive training for psychosis or supported employment or aerobic exercise) or with cognitive activation strategies. Exclusion criteria were as follows: papers that included different psychiatric disorders other than schizophrenia; papers not published in the English language; meta-analytic or systematic review studies; papers using different brain stimulation modalities poorly applied in PLWS (including transcranial alternating current stimulation, tACS; transcranial random noise stimulation, tRNS; deep rTMS, electroconvulsive therapy, ECT, magnetic seizure therapy, MST; transcranial focused ultrasound, tFUS). As this is a critical review, the exclusion of available studies was made according to inclusion and exclusion criteria and was not based on qualitative analysis.

Critical and narrative reviews represent one of the most common forms of medical literature synthesis, aiming to provide a comprehensive and critical assessment of the available evidence and to offer valuable insight for future research. However, as the selection of the included literature did not follow a systematic procedure, given the structure and the aims of the present review, it should be noted that the studies present in the review may not represent the totality of the works investigating the explored topic.

## 3. Results

The initial search yielded 321 articles. After duplication removal and title and abstract inspection, 38 full-text records were assessed for eligibility. Finally, a total of 11 original studies were included in the present review. This demonstrated that there is a dearth of studies examining the feasibility of combining NIBS with EBPIs to improve functional outcomes and symptom severity in PLWS, especially negative and cognitive symptoms. Only three reports combined rTMS/iTBS with CR [113,114,115], and one study combined rTMS with FI [116]. On the other hand, most studies combined NIBS, especially tDCS, with cognitive activation (CA) to improve cognitive functioning [117,118,119,120,121,122,123,124]. Table 1 provides a summary of the included studies.

### 3.1. Combining NIBS with CA

CA consists of repeated practice sessions in which cognitive exercises/tasks focus on one or multiple cognitive domains to improve the associated functions [125], also providing generalizable results to non-trained cognitive domains [126]. However, despite some elements being similar to CR, CA is not an EBPI in the case of schizophrenia. This is mainly because CA’s effectiveness varies considerably between studies as a consequence of different CA protocols, designs, and methodologies [126,127]. In other words, unlike CR, CA is not a standardized and manualized approach for the treatment of cognitive impairment and clinical features of schizophrenia [127]. In fact, a CR intervention for PLWS must require four core elements [i.e., (1) cognitive exercise; (2) a trained therapist, with a basic understating of cognitive processes and how cognitive abilities affect everyday functioning, providing the training; (3) the development of problem-solving strategies; (4) the inclusion of generalization procedures to facilitate the transfer of the learned skills to everyday functioning [36,48,127] to be effective for cognitive and functioning improvements. However, these elements are not necessarily present in CA studies.

Despite these differences, a recent meta-analysis by Poppe et al., 2024 [127] assessed whether different NIBS modalities (deep and HF-rTMS, iTBS, tDCS, transcranial Random Noise Stimulation, and transcranial Alternating Current Stimulation) in combination with CA could be helpful to improve cognitive functions and clinical and functional outcomes in healthy subjects and in patients with neurological and psychiatric disorders. Among the 72 included studies, while most studies (n = 59) used tDCS as the NIBS approach, only a minority (n = 5) [117,119,122,123,124] applied tDCS combined with CA in schizophrenia. Taking together the results from all clinical samples, only the domain of learning/memory showed significant but small improvements when CA was combined with NIBS post-intervention, but not in the long term. Furthermore, the combined intervention did not significantly improve either other cognitive domains or symptom severity, suggesting that cognitive enhancement did not transfer to real-life functioning improvements [127]. Similarly, Burton et al., 2023 performed a meta-analysis study focusing on the combination of CA and tDCS in neurological and psychiatric disorders [128]; of the 15 included studies, only 2 evaluated the effects of combining tDCS with CA in PLWS [117,123]. While most studies delivered tDCS to the left DLPFC, the pulled results showed a small but significant effect of the combined treatment on measures of attention and working memory, indirectly confirming the involvement of the left DLPFC in these cognitive domains [128]. Although these meta-analyses used a trans-diagnostic approach, the main limitations come from having included different clinical samples, heterogeneous study designs, mixed CA methodology (i.e., online versus offline training paired with stimulation sessions), and a relatively small number of studies to analyze cognitive outcomes [127,128].

In the context of schizophrenia, among the reviewed studies, the most solid results come from the largest trial by Orlov et al., 2017 [117]. In this double-blind sham-controlled study, 49 PLWS participated in 4 days (days 1, 2, 14, 56) of a non-standardized CA while concomitant 2 mA prefrontal-tDCS (anode: left DLPFC, cathode: right orbitofrontal region) was applied on day 1 and day 14 to verify if acute tDCS administration could facilitate performance on cognitive training tasks of working memory (using the n-back task) and implicit learning (using the implicit learning task) and if participants treated with active-tDCS would demonstrate improved retention of performance on the next day and on the longer-term follow-up. Moreover, it was assessed if the combined treatment could produce generalizable effects to on non-trained tasks on in other cognitive domains. While no acute improvement was found for any cognitive domain, the working memory task demonstrated significant performance improvement in the active-tDCS group at day-2 and at day-56 but not on implicit learning, suggesting that beneficial effects of tDCS on working memory required a consolidation period probably due to the tDCS mechanism of actions (i.e., LTP/LTD). However, only trend evidence of generalization onto untrained tasks on attention and vigilance tasks was found. Unfortunately, this study did not assess whether cognitive improvements could be generalizable to functional domains and real-world functioning. Subsequently, by using the same study protocol for CA and tDCS, Orlov et al., 2022 assessed if concurrent tDCS could improve learning rates on the stochastic sequence-learning task, confirming that performance improvements appeared not acutely after the stimulation but after a 24 h -consolidation phase following the tDCS administration (day-2) and that these improvements were maintained at the follow-up (day-56), suggesting long-lasting effects on learning [118]. Moreover, by using fMRI scans, the authors found that tDCS application was associated with increased brain activation in the insula and reduced activation in the amygdala in the active -tDCS group, while no direct effects were detected on the prefrontal cortex activity, suggesting that tDCS could modify the activation of deep brain structures involved in the neural circuits related to cognitive functioning [118].

Lo et al., 2022 provided the second-largest study in the field [119]. A total of 44 PLWS participated in a parallel-group, double-blinded, randomized controlled trial in which patients received either five sessions of concurrent 2 mA active tDCS (anode: left DLPFC) plus gamified CA (using a dedicated mobile app) or sham tDCS plus CA. Cognitive outcomes were assessed with the Cambridge Neuropsychological Test Automated Battery, Trail Making Test A, and Backward Digit Span (BDS) at three time points (baseline, after completion of the 5-day intervention, and at 4 weeks post-intervention). The authors found significant improvements in the working memory domain at the BDS immediately after the end of the intervention, as well as 4-weeks later, whereas no other cognitive dimension considerably improved. These results suggest that tDCS could promote synaptic plasticity, particularly in activated neuronal networks located in the left DLPFC during the practice of cognitive tasks in a manner that was state-dependent.

Nienow et al., 2019 [123] performed a single-blind, sham-controlled, randomized, proof-of-concept study in which a cognitive activation protocol was concurrently combined with 1 mA tDCS (anode: left DLPFC, cathode: right supraorbital position) in 10 PLWS. A cognitive activation protocol was provided in three 1-hour sessions weekly for 16 weeks. Training tasks included tasks selected from PSS CogRehab and BrainTrain’s Captain’s Log, as well as word and picture N-back tasks. tDCS was applied concurrently from the third week onward, during the first 20 min of cognitive training, performing two tDCS sessions each week (28 total). To evaluate simple learning, a non-adaptive word 2-back task was administered. To evaluate near transfer, a non-adaptive picture 2-back task was used. Distal transfer was measured with the overall composite score from the MATRICS Consensus Cognitive Battery (MCCB). At the study’s end, the authors found that, compared to sham treatment, active tDCS resulted in word 2-back and picture 2-back d task improvements; additionally, the MCCB overall composite improved in favor of the active treatment.

Weickert et al., 2019 [124] performed a randomized, double-blind, sham-controlled, parallel groups trial, investigating the simultaneous combination of spatial working memory training with 2 mA tDCS in 12 PLWS. A total of 20 tDCS sessions (anode: right DLPFC, cathode: left tempo–parietal junction) were performed daily over 4 weeks concurrently with a 2-back spatial working memory test (performed during the 20 minutes of each tDCS session). Cognitive improvement was assessed with the MATRICS Consensus Cognitive Battery (MCCB). The authors found that tDCS significantly improved language-based working memory after 2 weeks and verbal fluency after 2 and 4 weeks.

Congruently, the lack of positive effects on cognitive domains other than working memory was demonstrated by Schilling et al., 2022 [120]. In a randomized, double-blind, sham-controlled study on 36 PLWS, 12-daily sessions of 2 mA prefrontal-tDCS (anode: left DLPFC, cathode: right orbitofrontal region) combined with 15 min sessions of concurrent CA (by using the Cogniplus program to foster working memory, response inhibition and planning abilities) revealed no significant effects on other executive functions except for working memory (measured using reaction time changes at the n-back task) and divided attention (measured using the WAF battery) [120].

Despite positive effects on the working memory domain observed in previous studies, Shiozawa et al., 2016 designed a 5-day protocol in which 10 PLWS were randomly assigned to 10 sessions twice daily of either 2 mA anodal or sham prefrontal tDCS (anode: left DLPFC, cathode: right DLPFC) combined with CA consisting of working memory (n-back) and sequence learning tasks. However, the authors failed to demonstrate that the concomitant use of tDCS and CA could improve any cognitive outcomes [121].

Chang et al., 2021 [122] was not considered in our analysis as this study investigated another NIBS modality, the tACS, and this technique is only investigational in PLWS and beyond the scope of the present review.

### 3.2. Combining NIBS with EBPIs

Focusing on the studies that properly combined NIBS with EBPIs, while the most frequently applied psychosocial intervention was CR, discordant results were obtained [113,114,115]. Thirthalli et al., 2022 [113] delivered CR daily for 3 weeks and thereafter once a week for a total period of 4 months. Twenty-eight participants were randomized to receive either active (n = 15) or sham (n = 13) rTMS just prior to the delivery of CR sessions. Comparisons were made with treatment-as-usual (TAU) controls (n = 12). A 20 Hz neuronavigated-rTMS targeted the bilateral DLPFCs was provided over 3 weeks, with 100% of the resting motor threshold for 25 trains of 2 s with an inter-train interval of 30 s (1000 pulses per hemisphere). Comparing the CR + rTMS groups with TAU, significant group x time interaction effects were observed for executive functions, processing speed, and verbal learning domains, suggesting that greater cognitive improvements occurred in the CR + rTMS than in the TAU group. However, when the active versus sham-rTMS groups were compared, the results indicated relatively better performance in the sham-TMS group, suggesting that rTMS did not provide additional value [113].

Considering preliminary findings on the therapeutic role of iTBS targeting the left DLPFC to improve social cognition in PLWS [129], the single-blind study by Vergallito et al., 2024 was the first to apply iTBS as a primer with the aim of enhancing the effect of individualized training on neurocognitive abilities and social cognition [114]. In this pilot single-blind study, as 21 participants were randomized into four groups, the primary aims were to assess the impact of iTBS and cognitive training combination (active-iTBS + training, n = 6) on social cognition, comparing this approach to iTBS only (iTBS + no training, n = 7), cognitive training only (sham-iTBS + training, n = 5), or no intervention (sham-iTBS + no training, n = 3). Over 3 weeks, participants received 15 daily sessions of active or sham neuronavigated stimulation (20 iTBS trains, 600 pulses per session at 100% of active motor threshold) followed, or not, by 50-min of individualized training on nonsocial cognitive domains (first 30-min of the session to address attention, verbal and visual learning, processing speed, executive functions) and social cognition domain (remaining 20-min of the session to address emotion recognition, theory of mind, social perception and attributional bias). For the non-social training, a computerized version of CR, namely the COGPACK software, was used. For social cognition training, the Social Cognition Individualized Activities Lab (SoCIAL) was applied [129], with the first seven sessions dedicated to the Emotion recognition module and the following eight sessions for the Theory of mind module. Primary outcomes were assessed at the end of the treatment and at 1, 3, and 6 months follow-up with the Mayer-Salovey-Caruso Emotional Intelligence Test—Managing Emotions (MSCEIT-ME), Facial Emotion Identification Task, Awareness of Social Inference Test, and the Ambiguous Intentions Hostility Questionnaire. Preliminary findings from this pilot study showed negative results. Only a trend toward the improvement of emotional intelligence at the MSCEIT-ME was found in individuals participating in the training, while no changes over time were observed for the theory of mind, emotion recognition, and attributional bias scores. Thus, the authors concluded that iTBS did not provide significant effects on individuals’ performance as a standalone treatment nor when combined with CR [114]. Two main reasons could explain these negative findings. On the one hand, the fact of stimulating the DLPFC instead of other brain regions more clearly involved in the social cognition domain (i.e., the temporoparietal regions) [1,2]; on the other hand, it was noted that SoCIAL intervention provided only preliminary although effective results at improving social cognition abilities [130].

Subsequently, Vergallito et al., 2024 [115] applied the same methodology and sample used in the previous study but, in this case, the primary outcomes evaluated if the combination of CR and iTBS could improve cognitive impairments (assessed with the MATRICS Consensus Cognitive Battery, MCCB) and negative symptoms (assessed with Brief Negative Symptom Scale, BNSS) in PLWS. Again, negative results were found. On the one hand, as stand-alone interventions, iTBS and CR effectively improved negative symptoms and verbal learning and vigilance, respectively. In detail, iTBS produced a mild decrease in negative symptoms at the BNSS, but such improvements were not confirmed at the follow-up. CR produced significant improvements in verbal learning after the 3-week course and at the 6-month follow-up, while the improvement in the vigilance domain emerged only at the 6-month follow-up. On the other hand, the authors found that the combined intervention, active-iTBS + CR, did not provide additional benefits and failed to boost clinical improvements in PLWS. Nevertheless, it is worth noting that both negative findings by Vergallito [114,115] should be interpreted with caution as the authors presented only preliminary results driven by a very small sample for each treatment condition.

Finally, Li et al., 2020 performed the largest study in the field, hypothesizing that the combination of rTMS and FI would result in synergetic effects at improving clinical symptoms (assessed with Positive and Negative Syndrome Scale, PANSS) and cognitive functioning (assessed with the MCCB) [116]. This randomized controlled trial involved 200 first-episode drug-free PLWS, of which 188 completed the study, and 50 healthy controls. Patients were assigned randomly for a period of 12 weeks to receive treatment with risperidone only (risperidone group), a combination of rTMS and risperidone (rTMS group), FI + risperidone (family intervention group), or TMS + risperidone + FI (combined group). Each treatment condition included 47 patients. HF-rTMS protocol consisted of 20-min at 10 Hz over the left DLPFC at 80% of the motor threshold (4-sec trains, 40 pulses, intertrain interval of 56 s) for 10 daily sessions over 2 weeks. FI was a psychosocial intervention that included supervision regarding lifestyle, training in social skills, social functioning and social communication, and dedicated family support. The risperidone dosage was titrated from 1 to 4–6 mg/day. At the end of the study, significant decreases in PANSS scores and increases in MCCB scores in all groups were observed. In detail, PANSS negative subscale scores in the combined group and rTMS group were lower than those observed in the risperidone-only group. Moreover, for all PANSS subscales scores, the combined group showed the highest rate of participants with high response and the lowest rate of participants with low response compared to the other groups, suggesting that combined treatment most effectively improved clinical features of PLWS. Furthermore, contingency analysis revealed that the PANSS negative subscale scores were strongly correlated with allocation, indicating that the combined treatment produced a larger improvement in negative symptoms compared to rTMS. For cognitive symptoms, after 12-weeks, MCCB scores (attention/vigilance, working memory, and visuospatial Memory) with the combined treatment emerged as significantly superior to those observed in the other groups. The combined group had the highest rate of participants with a high level of response for MCCB scores, while the risperidone-only group showed the lowest rate of participants with a high level of response. Thus, highlighting that the duration of the combined treatment should last at least 12 weeks, it was suggested that the combined therapy produced the largest effects on clinical features and that rTMS and the FI were next in effectiveness. In conclusion, as remarkable synergistic effects were observed between rTMS and FI, a theoretical foundation for the provision of this combined therapeutical approach was proposed: while rTMS could increase neuron excitability and the activity of the prefrontal cortex, FI could reinforce these changes and further increase the synaptic connections and plasticity [116].

## 4. Discussion

Recommendations on the treatment of schizophrenia clearly suggested providing an integrated and multimodal approach combining pharmacological and non-pharmacological interventions to effectively improve functional outcomes and clinical features. As both EBPIs and NIBS represent feasible treatment options targeting the clinical features that are unmet needs for PLWS (such as negative symptoms, cognitive impairment, and psychosocial functioning), this critical review is the first to collect current evidence on the combination of these two non-pharmacological approaches. Despite several theoretical backgrounds suggested that combining psychological, cognitive, and behavioral interventions with NIBS could be a promising multimodal approach to improve treatment outcomes of several psychiatric disorders [131,132,133,134], our review demonstrated that only a few studies have combined NIBS and EBPIs in schizophrenia leading to the conclusion that this field of study is currently in its very infancy. Surprisingly, we found that no study implemented NIBS with commonly used psychosocial interventions, such as SST or CBTp. This is an important lack, especially for psychological interventions, namely CBTp. As both psychotherapy and NIBS can be tailored to the person (the former is highly personalized by definition while the latter can be personalized in terms of parameters, target, and type of stimulation), this combined approach could further represent a form of treatment hyper-personalization, as seen in many psychiatric disorders [131,132,133,134]. Thus, a suggestion is that future studies will combine NIBS with psychotherapy to additionally improve clinical outcomes also in the case of schizophrenia.

Conversely, CR was the most implemented EBPI though NIBS, especially with rTMS/iTBS, although inconclusive results were obtained on the effectiveness of such combination at improving negative symptoms and cognitive impairments in PLWS [113,114,115]. On the other hand, more solid findings derived from the combination of HF-rTMS with FI suggest that the integration of NIBS and FI could synergize with each other to improve treatment outcomes, including cognitive and negative symptoms [115]. However, as these findings come from only one RCT, further evidence is needed to confirm the synergic effects of combining HF-rTMS and FI. Additionally, promising results come from the combination of tDCS and CA on cognitive enhancement, especially in the working memory domain [118,119,123,124]. However, as previously mentioned, CA does not represent an established evidence-based intervention for PLWS. Anyway, the most important limitation of the available literature is that no study assessed whether the combination of EBPIs and NIBS could produce not only meaningful variations of clinical symptoms but also transfer to improvements in everyday psychosocial functioning. Thus, the general lack of functional outcome assessments is an important issue that future studies must consider, providing clear evidence of the effectiveness of such a combination.

Several reasons could be considered to understand the lack of effectiveness of the combination of these non-pharmacological treatments. The first issue is related to the choice of NIBS modality to be applied in clinical trials; in other words, which is the best NIBS approach to combine with EBPI? In our review, most studies applied rTMS/iTBS with CR or FI, while tDCS was only combined with CA. Although no definitive guidelines are available on the treatment of cognitive and negative symptoms via NIBS, a recent meta-analysis suggested that tDCS could be more effective than rTMS in improving clinical features of schizophrenia, especially for cognitive impairments [71]. Moreover, it is worth noting that, for the treatment of negative symptoms, the expert panel guidelines reduced recommendations on HF-rTMS to the left DLPFC from Level B (“probably effective”) [135] to Level C (“possibly effective”) [59]. Furthermore, the EPA guidelines on the treatment of cognitive symptoms did not recommend rTMS/iTBS as an evidence-based intervention, according to the results of several meta-analyses [70,96,97,98,99,100]. Thus, it is not surprising that the combination of EBPIs with rTMS/iTBS was not clearly associated with meaningful clinical variations, as seen in our review [113,114,115]. Moreover, when considering rTMS parameters that could influence clinical outcomes, meta-analysis studies suggested that to improve cognitive impairment in PLWS, stimulation should target the left DLPFC [96,101] with an intensity of 20 Hz [96]. However, even by considering such parameters, the reviewed studies provided divergent results. Indeed, while Li et al., 2020 clearly demonstrated that 20 Hz rTMS was successfully combined with FI, providing meaningful variations in negative and cognitive symptoms, Thirthalli et al., 2022 did not find significant improvement in cognitive symptoms when 20 Hz rTMS was combined with CR. On the other hand, as tDCS was delivered concurrently with CA only rather than in combination with a proper EBPI, it is not possible to provide specific recommendations on such a combination. Nevertheless, we suggest that future clinical trials will consider combining an EBPI with tDCS as this NIBS modality is inexpensive, portable, and associated with a lower risk of adverse events than other NIBS techniques. Moreover, tDCS application is more silent and usually produces a momentary tingling/itching sensation, resulting in fewer distractions compared to rTMS/iTBS, in which stimulation produces an audible clicking noise and somatic sensations related to contractions of scalp muscles and peripheral nerve excitation. All these factors make tDCS an ideal tool for studying its combination with an EBPI, especially with psychotherapeutic interventions [134].

The second relevant issue is the timing of the combination between an EBPI and NIBS. In other words, NIBS sessions could be performed as a primer (a sequential application where NIBS are applied before EBPI sessions, producing addictive effects) or concurrently with psychosocial/behavioral training sessions (a simultaneous application of NIBS and an EBPI producing synergistic effects). Research from experimental neuroscience highlighted that the NIBS’s effects are state-dependent, meaning that the condition of the cortical region targeted during the stimulation has a significant impact on the NIBS’s effects on cortical excitability and behavioral outcomes [131,134,136,137,138,139]. In this context, researchers hypothesized the concept of functional targeting, which involves combining a cognitive task or intervention in order to activate specific neural circuits and concurrently targeting those same circuits with NIBS [134]. As a consequence, the state-dependency effects of NIBS could be leveraged and lead to the development of multimodal interventions by combining behavioral activation with NIBS [134]. Based on these concepts, researchers usually applied rTMS as a primer, considering its mechanism of action, its more focal effects, and higher spatial resolution compared to tDCS [124,134]. Conversely, tDCS was suggested to be performed simultaneously in combination with behavioral/cognitive training, producing synergistic neuro-enhancing effects with the idea that a lower spatial resolution could be responsible for the stimulation of larger cortical networks rather than one specific brain area, as in the case of rTMS [124,134]. Our review confirmed such evidence. In fact, for all studies that combined tDCS with CA, the electrical stimulation was performed simultaneously with CA, and in these cases, the main findings were a significant improvement in the working memory domain [36,117,118,119,127]. Nevertheless, despite positive evidence on the combination of CA and tDCS at improving cognition in PLWS, evidence is too limited to establish if tDCS should combined with CA concurrently on a single-day stimulation session rather than over multiple days (with a sequential daily application over subsequent days) to obtain greater and more durable neuroplastic effects and cognitive performance improvements [131]. In fact, on the one hand, a meta-analysis study, including prefrontal, single-session tDCS studies to evaluate the effects on cognitive performance, showed that in individuals with mental health disorders, concurrent tDCS + CA produced greater effects on cognitive performance compared to a sequential task administration [140]. On the other hand, the evidence collected in our review included divergent tDCS protocols (single-session studies [117,118] *versus* studies with once-daily stimulation over multiple days [119,120,121] *versus* studies with twice-daily stimulation [123]), leading to the conclusion that for PLWS, it is not clear if tDCS should be applied concurrently with a single session or over multiple days. Additionally, as neuroscience researchers established that tDCS could also be used as a primer due to its metaplastic-like phenomena [141], we suggest that further studies will assess the feasibility of combining an EBPI and tDCS as a primer in PLWS.

Conversely, those studies that combined rTMS/iTBS with CR performed this NIBS modality as a primer (just before the CR sessions), confirming the suggestions above. However, in this case, rTMS/iTBS did not provide additional value on cognitive and negative symptoms in PLWS [113,114,115]. A reason that could explain these negative findings could be related to the fact that studies in which rTMS/iTBS and CR were combined [113,114,115] included a very low sample size. Thus, it is too premature to definitively conclude that these NIBS modalities cannot be effectively used as a primer in the case of schizophrenia since more solid findings are needed.

Moreover, as no dedicated comparative studies (simultaneous versus primer approach) are available, no definitive conclusion can be provided regarding this issue. Direct comparison studies may represent an interesting perspective for future research in order to provide a definite answer regarding the ideal timing of the NIBS and EBPI combination. However, it could also be argued that a concurrent and simultaneous application might be more feasible in real-world rehabilitation contexts. On the other hand, implementing an EBPI with NIBS protocol as a primer could be very demanding for participants’ time and ability to attend both interventions, limiting the dissemination of this approach to inpatient settings [114,115]. Moreover, a sequential approach would be more complex as time and resource allocation for the service providing the program, again lowering its feasibility and practical acceptability.

By considering the EBPI modality, a third relevant issue that must be considered is related to CR protocols. As previously described, to be effective, a valid CR intervention must incorporate four core elements to produce significant cognitive and functioning improvements in PLWS. However, neither Vergallito et al. [114,115] nor Thirthalli et al., 2022 [113] lack a clear description of the fact that the CR intervention included the four core elements of CR. These methodological issues make the obtained results difficult to generalize and absolutely preliminary. Moreover, in Vergallito et al. [114,115], the CR protocol consisted of a modified procedure in which both neuro-cognitive and social cognition were implemented with dedicated training. In detail, the social cognition training was more intensive (e.g., every day for 3-weeks) if compared to other evidence [142], leading the authors to conclude that the negative results observed at the study end could be explained by considering that the provision of closer training sessions could prevent participants from retaining the acquired knowledge and putting them into practice. Therefore, it is necessary that future studies combining NIBS with CR will take into consideration that all the core elements that make the CR intervention effective are congruently applied and that CR is applied accordingly to guidelines, avoiding modifications.

Other issues that probably limit the acquisition of clear scientific evidence on the combination of NIBS and an EBPI in schizophrenia are linked, on the one hand, to the restricted diffusion of NIBS in public mental health care settings and, mostly, by the time commitment required to the patient [132]. Indeed, usually, NIBS requires multiple sessions of stimulation, performed daily over several weeks. However, this represents a significant barrier for those patients who cannot make a daily commitment [132]. A way to overcome these limitations could be to design accelerated tDCS protocols where multiple stimulation sessions will be performed in a single day or to combine an EBPI with iTBS as the latter has a short time of delivery. However, Vergallito and colleagues [114,115] clearly reported the difficulty of engaging PLWS in clinical trials that combined iTBS and CR. The main reasons for which PLWS denied participating in the studies were the lack of illness insight and the feeling that protocol was too demanding. Thus, despite the potential effectiveness of iTBS in treatment-resistant depression, for which there is solid evidence regarding the possibility of providing accelerated protocols [143], in the case of schizophrenia, unfortunately, the scientific literature does not provide definitive suggestions on how to reduce the timing of NIBS application. In fact, while for tDCS, only preliminary evidence is available on accelerated protocols feasibility with a frontotemporal electrode placement [144,145], for HF-rTMS/iTBS targeting the DLPFC or the cerebellum, more evidence suggested that twice daily stimulation protocols could significantly decrease negative symptoms and improve cognitive functioning in PLWS [146].

Another significant issue that should be taken into account and has not yet received significant attention in the available literature is the moderating effect of specific patient characteristics on the efficacy and effectiveness of combined interventions. This is particularly significant considering that schizophrenia represents a very heterogeneous disorder, and some clinical features, such as global symptoms severity, as well as the severity of positive and negative symptoms and the extent of cognitive impairment, can vary considerably from patient to patient. In regard to CR, the results of recent and comprehensive meta-analyses show that more clinically compromised participants obtain greater improvements in cognitive performance and psychosocial functioning [48,147,148]. Whether these moderator effects play a role in NIBS and EBPI combinations, however, remains to be investigated in the future with dedicated studies.

Beyond the combination of NIBS with CR, FI, and cognitive activation, it is worth noting that other EBPI modalities could be more easily applied, maybe with fewer burdens both for the patients and mental health providers. Among these EBPIs, physical exercise (particularly AE) represents a valid option due to its beneficial effects on positive, negative, and cognitive symptoms and also on physical health in general [47,48,149,150]. Moreover, thanks to its high practical feasibility, physical exercise seems to be extremely appealing for psychiatric rehabilitation [147]. Although this review demonstrated that no clinical study on the combination of physical exercise and NIBS is available, initial data showed promising results in PLWS on body weight and body mass index reduction following either accelerated cTBS over the left primary motor area [151,152] or HF-rTMS over the left DLPFC [153]. Thus, we advise that future studies should evaluate the feasibility of combining physical exercise and NIBS in PLWS, confirming the preliminary findings on NIBS’s effectiveness in improving physical and metabolic outcomes.

Finally, a limitation of the present critical review is that it followed a narrative approach, and therefore, it was not based on a systematic literature search and did not adhere to PRISMA recommendations for systematic reviews and meta-analyses. However, performing a systematic review and meta-analysis was beyond the scope of the present work, which instead aimed to provide a narrative and critical overview of the available literature. Moreover, numerous methodological issues may, at least at the moment, hinder the potential interest of a meta-analysis study. These problems concern the very limited number of studies combining NIBS and an actual EBPI, the heterogeneity of NIBS protocols, the different EBPIs used, the heterogeneity of clinical and cognitive outcomes, and adopted assessment measures. All these issues concerning the comparisons between studies would lead to a high level of heterogeneity of observed results and a low level of reliability of the conclusions.

## 5. Conclusions

Our review demonstrated that, for schizophrenia, the field of combining an EBPI and NIBS is in its infancy. In fact, only a few trials combined these non-pharmacological modalities to improve treatment outcomes in PLWS. While emerging evidence is available on the combination of CR and rTMS/iTBS, inconclusive results were obtained, suggesting the need to perform further well-designed studies. Conversely, albeit preliminary, more solid findings are available on the combination of HF-rTMS and FI to improve clinical features in PLWS. Moreover, although CA could not be considered an EBPI, promising results are available on the combination of tDCS and CA to improve cognitive impairment, especially in the working memory domain. Finally, no clear evidence is available on the efficacy of such a combination in improving psychosocial functioning in order to achieve recovery for PLWS.

By summarizing the available literature, our review also highlighted several methodological issues that must be considered to design further dedicated trials.

In conclusion, further efforts are needed to confirm whether NIBS and an EBPSI could be effectively integrated as multimodal interventions to improve treatment outcomes and achieve recovery in PLWS.

## Figures and Tables

**Table 1:** Summary of the included studies that combined NIBS with evidence-based psychosocial interventions or cognitive activation.

Authors [Reference]	NIBS Protocol				EBPI and CA	Sample	Outcomes
	Modality	Intensity	Stimulation Site	Duration (n° of Sessions and Frequency)			
Thirthalli et al., 2022 [113]	rTMS	20 Hz	Bilateral DLPFC	15 once daily	CR performed daily for 3 weeks once a week, and thereafter for 4 months	28 PLWS 12 TAU patients	Comparing the CR + rTMS groups with TAU, group x time interaction was significant for executive functions, speed of processing, and verbal learning, suggesting that cognitive performance improved better in the CR + rTMS than in the TAU group. When the active versus sham-rTMS groups were compared, a better performance in the sham-TMS group was found. The combined intervention (active-rTMS + CR) did not provide additional value.
Vergallito et al., 2024 [114]	iTBS	50 Hz	Left DLPFC	15 once daily	CR with COGPACK and SoCIAL	active-iTBS + training (n = 6) iTBS + no training (n = 7) sham-iTBS + training (n = 5) sham-iTBS + no training (n = 3)	Only a trend toward the improvement of emotional intelligence at the MSCEIT-ME was found in participants performing the training. No changes over time were found for emotion recognition, theory of mind, and attribution of intentions scores. iTBS did not provide significant effects on individuals’ performance as a standalone treatment nor when combined with CR.
Vergallito et al., 2024 [115]	iTBS	50 Hz	Left DLPFC	15 once daily	CR with COGPACK and SoCIAL	active-iTBS + training (n = 6) iTBS + no training (n = 7) sham-iTBS + training (n = 5) sham-iTBS + no training (n = 3)	As stand-alone interventions, iTBS and CR effectively improved negative symptoms and verbal learning and vigilance at the MCCB, respectively. The combined intervention, active-iTBS + CR, did not provide additional benefits, failing to boost clinical improvements.
Li et al., 2020 [116]	rTMS	10 Hz	Left DLPFC	10 once daily	FI	risperidone group (n = 47) rTMS + risperidone (n = 47) FI + risperidone (n = 47) rTMS + risperidone + FI (n = 47)	PANSS negative subscale scores in the rTMS + risperidone + FI group and rTMS group were significantly lower than those of the risperidone group. For all PANSS subscales scores, the rTMS + risperidone + FI group showed the highest percentage of high-response patients and the lowest percentage of low-response compared to the other groups. Contingency analysis revealed that the PANSS negative subscale scores were strongly correlated with the group: the rTMS + risperidone + FI group produced a larger improvement in negative symptoms than the rTMS group.
							The rTMS + risperidone + FI group had the greatest percentage of patients who showed a high response for MCCB scores, while the risperidone group had the lowest percentage of patients showing a high response. Remarkable synergistic effects between rTMS and FI therapy were observed.
Orlov et al., 2017 [117]	tDCS	2 mA	Anode: left DLPFC Cathode: right OFC	2 once daily	CA on working memory and implicit learning	49 PLWS	No acute improvement was found for any cognitive domain. The working memory task demonstrated significant improvements following active tDCS on day 2 and on day 56 but not on implicit learning.
Orlov et al., 2022 [118]	tDCS	2 mA	Anode: left DLPFC Cathode: right OFC	2 once daily	CA on stochastic sequence-learning task	49 PLWS	Performance improvements appeared not acutely after the stimulation but after a 24 h consolidation phase following the tDCS administration. Cognitive improvements were maintained at the follow-up suggesting long-lasting effects on learning.
Lo et al., 2022 [119]	tDCS	2 mA	Anode: left DLPFC Cathode: N/A	5 once daily	Gamified CA	44 PLWS	Significant improvements in the working memory domain at the Backward Digit Span immediately after the end of the intervention, as well as 4 weeks later, were noted. No other cognitive dimension considerably improved.
Schilling et al., 2022 [120]	tDCS	2 mA	Anode: left DLPFC Cathode: right OFC	12 once daily	CA (Cogniplus program)	36 PLWS	No significant effects on executive functions were noted except for working memory and divided attention.
Shiozawa et al., 2016 [121]	tDCS	2 mA	right DLPFC	10 once daily	CA on working memory and sequence learning tasks	10 PLWS	Concomitant use of tDCS and CT did not improve any cognitive outcomes.
Nienow et al., 2019 [123]	tDCS	1 mA	anode: left DLPFC, cathode: right OFC	28 Twice daily	PSS CogRehab and BrainTrain’s Captain’s Log plus working memory tasks (word and picture n-back tasks)	10 PLWS	Compared to the sham treatment, concurrent active tDCS resulted in word 2-back and picture 2-back d task improvements, leading to significant improvement of the working memory domain. Additionally, the MCCB overall composite improved in favor of the active treatment.
Weickert et al., 2019 [124]	tDCS	2 mA	Anode: right DLPFC Cathode: left TPJ	20 once daily	CA on working memory tasks (2-back spatial working memory task)	12 PLWS	Concurrent anodal tDCS significantly improved language-based working memory tasks after 2 weeks and verbal fluency tasks after 2 and 4 weeks at the MCCB battery.

CA: cognitive activation; CR: Cognitive Remediation; DLPFC: Dorsolateral Prefrontal Cortex; EBPI: evidence-based psychosocial interventions; FI: Family Intervention; Hz: hertz; iTBS: intermittent Theta Burst Stimulation; MCCB: MATRICS Cognitive Consensus Battery; MSCEIT-ME: Mayer-Salovey-Caruso Emotional Intelligence Test—Managing Emotions; mA: milli Ampere; MT: Motor Threshold; N/A: not applicable; PANSS: Positive and Negative Syndrome Scale; PLWS: people living with schizophrenia; TAU: treatment as usual; TPJ: temporo–parietal junction; rTMS: repetitive Transcranial Magnetic Stimulation; SoCIAL: Social Cognition Individualized Activities Lab; tDCS: transcranial Direct Current Stimulation.

## Data Availability

No new data were created or analyzed in this study.

## Abbreviations

ACT	assertive community treatment
AVH	auditory verbal hallucinations
AE	aerobic exercise
cTBS	continuous Theta Burst Stimulation
FC	functional capacity
CBTp	cognitive-behavioral therapies for psychosis
CT	cognitive training
CR	cognitive remediation
DBS	Backward Digit Span
DLPFC	dorsolateral prefrontal cortex
EBPI	evidence-based psychosocial intervention
EPA	European Psychiatric Association
FI	family interventions
GABA	Gamma-aminobutyric acid
HF-rTMS	high-frequency Transcranial Magnetic Stimulation
Hz	hertz
iTBS	intermittent Theta Burst Stimulation
LF-rTMS	low-frequency Transcranial Magnetic Stimulation
LTD	long-term depression
LTP	long-term potentiation
mA	milliAmpere
MCCB	MATRICS Consensus Cognitive Battery
MCT	Metacognitive training for psychosis
MSCEIT-ME	Mayer-Salovey-Caruso Emotional Intelligence Test—Managing Emotions
NIBS	Non-Invasive Brain Stimulation
NMDA	N-methyl-D-aspartate
PANSS	Positive and Negative Symptom Scale
PE	psychoeducation
PLWS	people living with schizophrenia
QoL	quality of life
rTMS	repetitive Transcranial Magnetic Stimulation
SE	supported employment
SGA	second-generation antipsychotics
SoCIAL	Social Cognition Individualized Activities Lab
SST	Social Skill Training
TAU	treatment as usual
tDCS	transcranial Direct Current Stimulation
UPSA-B	University of California, San Diego Performance-Based Skills Assessment
